# Electrochemical Mechanism of Molten Salt Electrolysis from TiO_2_ to Titanium

**DOI:** 10.3390/ma15113956

**Published:** 2022-06-02

**Authors:** Xianghai Meng, Hongmei Zhao, Sheng Bi, Zilai Ju, Zhenming Yang, Yu Yang, Hui Li, Jinglong Liang

**Affiliations:** 1 Department of Mechanical Engineering, Tangshan Polytechnic College, Tangshan 063299, China; xianghaimeng@163.com (X.M.); tsgzyzhm523721@163.com (H.Z.); b_shinney@126.com (S.B.); jzl-sky@163.com (Z.J.); sciwztg@163.com (Z.Y.); 2 Key Laboratory of Ministry of Education for Modern Metallurgy Technology, College of Metallurgy and Energy, North China University of Science and Technology, Tangshan 063210, China; 18332602809@163.com (Y.Y.); lh@ncst.edu.cn (H.L.)

**Keywords:** TiO_2_, molten salt, electrochemical reduction, electrochemical mechanism

## Abstract

Electrochemical mechanisms of molten salt electrolysis from TiO_2_ to titanium were investigated by Potentiostatic electrolysis, cyclic voltammetry, and square wave voltammetry in NaCl-CaCl_2_ at 800 °C. The composition and morphology of the product obtained at different electrolysis times were characterized by XRD and SEM. CaTiO_3_ phase was found in the TiO_2_ electrochemical reduction process. Electrochemical reduction of TiO_2_ to titanium is a four-step reduction process, which can be summarized as TiO_2_→Ti_4_O_7_→Ti_2_O_3_→TiO→Ti. Spontaneous and electrochemical reactions take place simultaneously in the reduction process. The electrochemical reduction of TiO_2_→Ti_4_O_7_→Ti_2_O_3_→TiO affected by diffusion was irreversible.

## 1. Introduction

Titanium is considered a rare metal because it is dispersed in nature and difficult to extract. However, it is relatively abundant, ranking tenth among all elements. Titanium ore mainly ilmenite and rutile, widely distributed in the earth’s crust and lithosphere. Titanium and its alloys have been widely used in aerospace, national defense, ocean, energy, transportation, medical, and other fields due to its advantages of low density, high specific strength, good heat resistance, and corrosion resistance [1,2,3]. Therefore, titanium has a “21st century metal”, “all-round metal”, and “modern metal” reputation [4].

Due to titanium and oxygen, nitrogen, carbon, hydrogen, and other elements have a strong affinity, making the titanium production process complex, a long process with high energy consumption and high cost, limiting the application of titanium in many industries. In order to reduce the production cost of titanium, researchers continue to improve the traditional process and develop new extraction methods. At present, Kroll process is the most important industrial process for titanium production. However, the complex process, long process, high energy consumption, and high cost limit the application of titanium in many industries [5,6]. In order to reduce the production cost of titanium, researchers have developed many new processes, among which the molten salt electrolysis method has attracted a lot of attention worldwide because of its characteristics of short process, low energy consumption, and simple process [7,8,9,10,11,12]. Using alkaline metal or alkaline earth metal salt as electrolyte, TiO_2_ as cathode, and graphite as anode, titanium was prepared by direct electrodeoxidation of TiO_2_ in the molten salt electrolysis method. Titanium can be obtained in one-step reduction process [13,14]. At present, the electrochemical method has already been intensely studied in preparation of alloys [15,16,17,18,19] and carbides [20].

In order to clarify the deoxidation process of TiO_2_ in molten salt electrolysis, the preparation of titanium by direct electro-deoxidation of TiO_2_ in NaCl-CaCl_2_ binary molten salt system was carried out in this work. The reduction process and electrochemical mechanism of the molten salt electrolysis from TiO_2_ to titanium were studied by potentiostatic electrolysis and electrochemistry analysis in detail.

## 2. Experimental Procedures

### 2.1. Raw Materials and Cathode Precursor Preparation

TiO_2_ (96 wt.%) and carbon (4 wt.%) powders of 2 g were used as raw materials and mixed homogeneously. The mixed powders were die-pressed at 20 MPa in a cylindrical mold (30 mm in diameter). The die-pressed bodies were sintered at 353 K for 8 h; then, the sintered disc was tied in the titanium electrode rod with a nickel wire as a cathode.

### 2.2. Electro-Deoxidation Process

**Figure 1:**
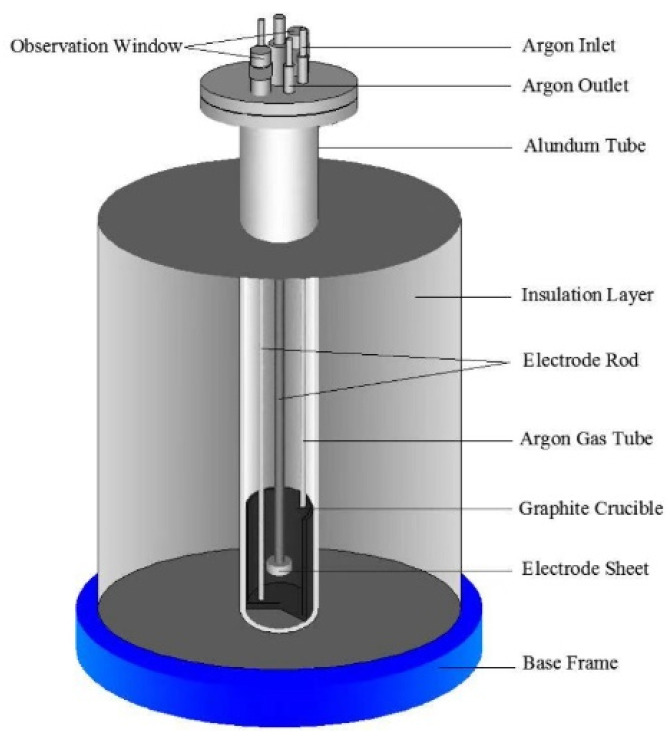
Schematic diagram of the electrolysis experimental device.

Anhydrous NaCl and CaCl_2_ salt (500 g in molar ratio 0.48:0.52) were placed in graphite crucible and dried in the steel reactor at 473 K for 8 h to remove moisture in the salt. When the molar ratio was NaCl:CaCl_2_ = 0.48:0.52, the lowest eutectic temperature point of the binary salt was 762 K [21]. In order to ensure that the molten salt system has low viscosity and high conductivity, the temperature conducted for this experiment is 1073 K. Then the temperature of the binary salt was programmatically raised in the reactor to 1073 K, while argon was continuously pumped into the reactor. The anode was graphite crucible, which was connected by a titanium electrode rod. The electro-deoxidization experiment was conducted at a constant potential of 3 V for 6 h. The schematic diagram of the experimental device was shown in Figure 1. The obtained cathodic products were washed by deionized water in the ultrasonic cleaners and vacuum dried at 333 k.

### 2.3. Electrochemical Test

**Figure 2:**
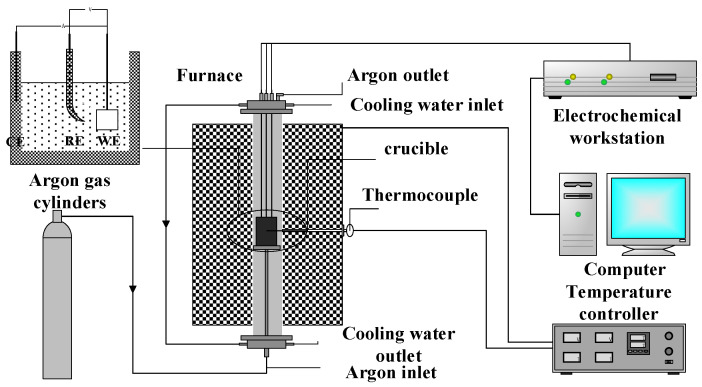
Schematic diagram of the electrochemical experimental platform.

The electrochemical deoxidation process from TiO_2_ to titanium was evaluated in a three-terminal electrochemical cell by PARSTAT 2273 electrochemical workstation. Pt wire (99.99%, *φ* = 0.5 mm), Mo wire (99.99%, *φ* = 0.5 mm), and graphite crucible were used as the reference, work, and counter electrodes, respectively. Cyclic voltammetry (CV) and square wave voltammetry were used to analyze the reduction of TiO_2_ to titanium in NaCl-CaCl_2_ at 800 °C. The schematic diagram of the experimental platform is shown in Figure 2.

### 2.4. Characterization

The electrolytic voltage was supplied by DC power supply (DP310, MESTEK, China). The phase composition of the solid precursors and cathodic products were determined by X-ray diffraction (XRD) (Noran7, Thermo Fisher, Waltham, MA, USA). Each scan was 5°–90° and step size is 0.02°. The morphology and chemical composition of the solid precursors and cathodic products were characterized by scanning electron microscopy (SEM) (S-4800, Hitachi, Tokyo, Japan) and energy dispersive X-ray spectroscopy (EDX). The acceleration voltage of SEM is 20 kV and the working distance (WD) is 10 mm.

## 3. Results and Discussion

### 3.1. Calculation of the Theoretical Decomposition Potentials

**Figure 3:**
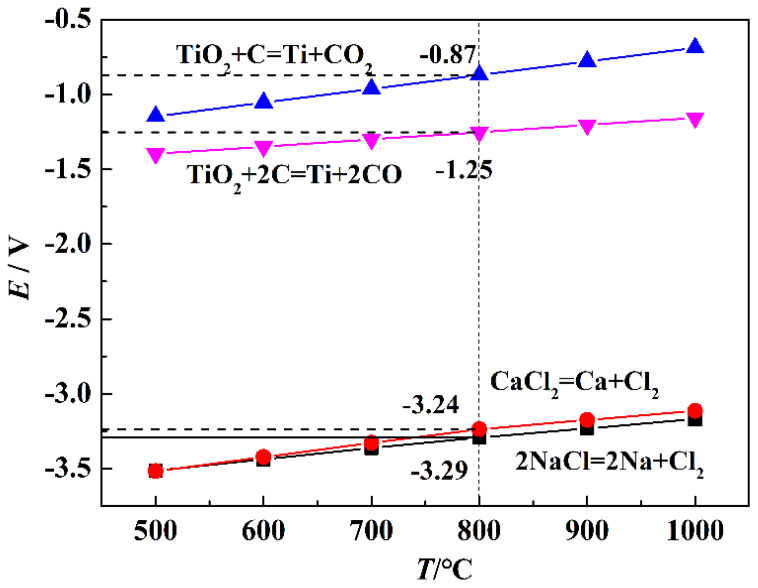
Theoretical decomposition potentials and reactions occurred in the electro-deoxidation cell from 773 K to 1273 K.

Alkaline metal molten salts with low melting point, wide electrochemical window, and good electrical conductivity are commonly used as electrolytes for electrochemical preparation of metals. The Gibbs free energy of the possible reactions can be calculated by HSC thermodynamics software. The theoretical decomposition potentials (*E*) of the metal molten salts and TiO_2_ were calculated by the following equation [22,23]:

(1) $$E=\frac{-\Delta G^{\Theta }}{nF}$$

where Δ*G*^Θ^ (kJ/mol) is the standard Gibbs free energy change; *n* and F represent the electron transfer number and Faraday’s constant (96,485 C/mol), respectively. The theoretical decomposition potentials and reactions that occurred in the electro-deoxidation cell from 773 K to 1273 K are listed in Figure 3. The results show that the theoretical decomposition potentials of TiO_2_ and the binary salt are positively correlated with temperature. The theoretical decomposition potentials of NaCl and CaCl_2_ is −3.29 V and −3.23 V, respectively, which is much higher than that of TiO_2_. It indicates that the experiment voltage of 3 V, conducted in a two-electrode system, is sufficient to electro-deoxidize TiO_2_ to titanium without the electrolyte decomposition.

### 3.2. Electro-Deoxidization of the Cathode Precursor

**Figure 4:**
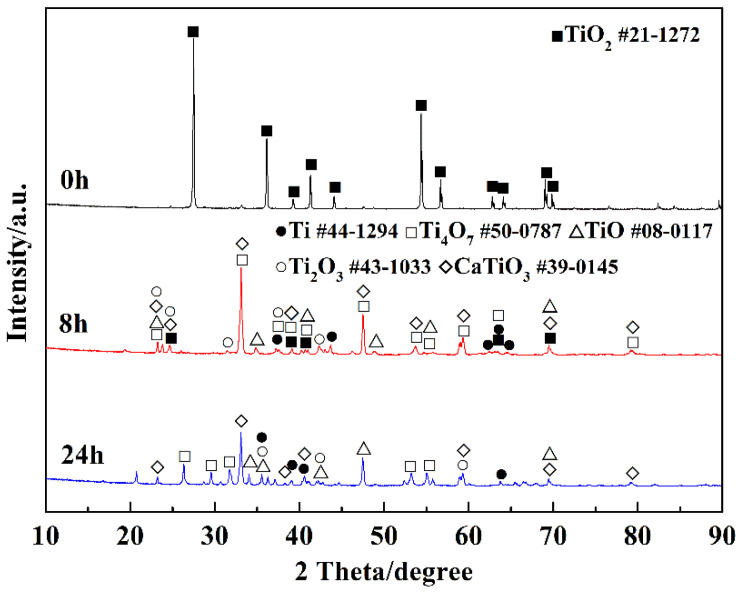
XRD patterns of the products at different electro-deoxidation times.

**Figure 5:**
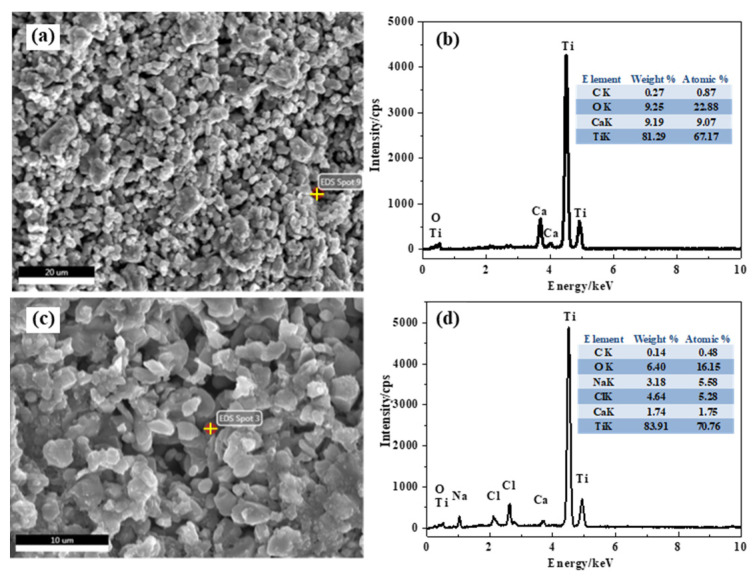
SEM images and EDX analysis of the products electrolysis for (**a**,**b**) 8 h and (**c**,**d**) 24 h.

Figure 4 presents the XRD patterns of the products at different electro-deoxidation time. It can be seen from the product electrolyzed for 0 h that TiO_2_ is the main component of the cathode precursor, which indicates that the little carbon did not react with TiO_2_ in the sintering process. The product electrolyzed for 8 h shows the intermediate valence titanium oxides (Ti_4_O_7_, Ti_2_O_3_, TiO) and CaTiO_3_ are the main phases of the product after 8 h electrolysis. CaTiO_3_ is generated by the reaction between TiO_2_ and calcium ions in molten salt and oxygen ions extracted from TiO_2_. Table 1 lists the possible reaction Δ*G*^Θ^ in the electrolysis process at 1073 K. Reaction (1) has an extremely negative Δ*G*^Θ^(−1045.43 kJ/mol) at 1073 K, indicating that the formation of CaO betweent Ca^2+^ and O^2−^ extracted from TiO_2_ is easy to proceed. The Δ*G*^θ^ of CaTiO_3_ generated by the reaction of CaO and TiO_2_ was −86.94 kJ/mol, demonstrating that the reaction could occur spontaneously. Literatures show that there is a high concentration of oxygen in the material at this stage; that is, CaTiO_3_ will be spontaneously formed when calcium ions and oxygen ions existed in the molten salt [24]. The diffraction peak of titanium detected in the product electrolyzed for 8 h indicates that titanium metal can be reduced after 8 h of electrolysis. Compared with the product of electrolysis for 8 h, the diffraction peak of titanium in the product of electrolysis for 24 h is significantly increased (shown in the XRD pattern of the product electrolyzed for 24 h), indicating that the reduction of titanium metal is further carried out with the extension of the electrolysis time. Figure 5 presents SEM images and EDX analysis of the products electrolysis for 8 h and 24 h. Combined with XRD data analysis in Figure 4, they show that CaTiO_3_ was formed in the products electrolysis for 8 h during the electrolysis process, shown in reaction (2). The main phase is the intermediate valence titanium oxides, and the CaTiO_3_ phase almost disappears in the products electrolysis for 24 h, which is due to the spontaneous decomposition between CaTiO_3_ and titanium, shown in reaction (3). The deposited carbon can react with the metal on the cathode, resulting in high carbon content in the cathode product. It can be explained by the following two reactions.

In anode:

$${\mathrm{CO}}_{2}+O^{2-}={\mathrm{CO}}_{3}^{2-}$$

In cathode:

$${\mathrm{CO}}_{3}^{2-}+4e^{-}=C+3O^{2-}$$

**Table 1:** Δ*G*^θ^ of possible reaction in the electrolysis process at 1073 K.

Possible Reactions	Δ*G*^θ^_1073 K_ (kJ/mol)	No.
Ca^2+^ + O^2−^ = CaO	−1045.43	(1)
CaO + TiO_2_ = CaO·TiO_2_	−86.94	(2)
Ti + CaTiO_3_ = 2TiO + CaO	−21.29	(3)

### 3.3. Electro-Deoxidation Thermodynamics of Titanium Oxides in Molten Salt Systems

The main phases in TiO_2_ electro-deoxidation products include Ti_4_O_7_, Ti_2_O_3_, TiO, and Ti. When graphite was used as the anode material, the main anode product in molten salt electrolysis was CO_2_ [25]. In order to simplify the calculation, CO_2_ was considered as the only gas component in the anode product. Table 2 listed Δ*G*^θ^ and *E* of TiO_2_ electro-deoxidation reactions at 1073 K. The theoretical decomposition potentials of TiO_2_ deoxidized to Ti_4_O_7_ is 0.34 V, which is lower than TiO_2_ deoxidized to Ti_2_O_3_, TiO, and Ti. Therefore, the reaction (4) is preferentially carried out under the voltage driving force, and the first step reaction controlled by electrochemistry produces Ti_4_O_7_ [26].

Table 3 listed Δ*G*^θ^ and *E* of Ti_4_O_7_, Ti_2_O_3_, and TiO electro-deoxidation reactions at 1073 K. The results show that *E* of Ti_4_O_7_ deoxidized to Ti_2_O_3_ is 0.48 V, which is lower than Ti_4_O_7_ deoxidized to TiO and Ti. Therefore, the second step reaction controlled by electrochemistry was Ti_4_O_7_ deoxidized to Ti_2_O_3_. In the same way, the third step reaction was Ti_2_O_3_ deoxidized to TiO. Finally, TiO deoxidized to Ti. According to the products obtained at different electrolysis times and electro-deoxidation thermodynamics analysis, the molten salt electrolysis from TiO_2_ to titanium is a multi-step electrochemical reaction process, which can be summarized as: TiO_2_→Ti_4_O_7_→Ti_2_O_3_→TiO→Ti.

### 3.4. Analysis of Electrochemical Deoxidation of TiO_2_ in NaCl-CaCl_2_ System

**Figure 6:**
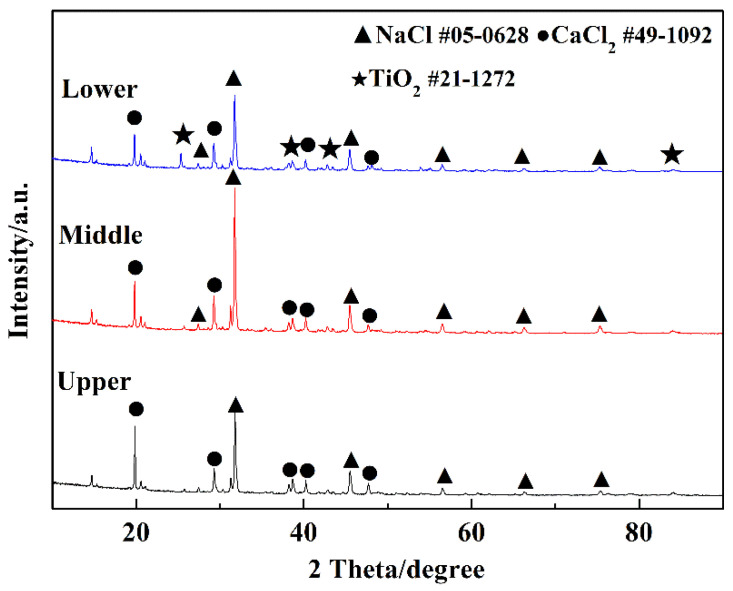
XRD patterns of the binary salt samples taken from upper, middle, and lower crucibles.

Then, 3 wt.% TiO_2_ was added to NaCl-CaCl_2_ binary molten salt system, and then the samples from the upper, middle, and lower crucibles were taken for XRD analysis after being heated to 1073 K for 4 h. The XRD patterns (Figure 6) show that no other substances were found in the samples taken from the upper and middle crucibles and TiO_2_ was deposited in the bottom of the crucible. It indicates that there is no chemical dissolution of TiO_2_ in the molten salt system. CaTiO_3_ cannot be formed spontaneously, because there is no electro-deoxidation reaction conducted to produce oxygen ions in the binary molten salt system.

**Figure 7:**
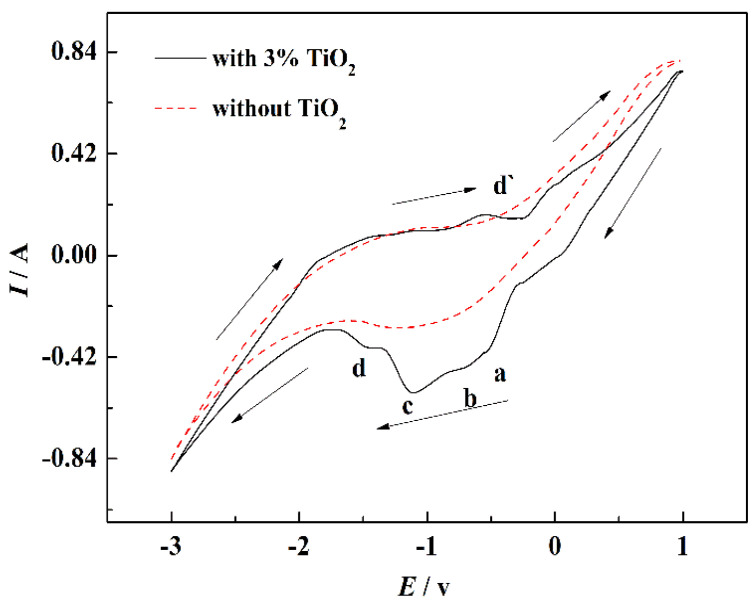
CV curves of NaCl-CaCl_2_ system before and after 3 wt.% TiO_2_ addition with 0.7 V/s vs. Pt scan rate.

Figure 7 displays the CV curves of NaCl-CaCl_2_ system before and after TiO_2_ addition. There is no redox peak found in the CV curve of NaCl-CaCl_2_ system without 3 wt.% TiO_2_; it demonstrates that the electrochemical properties of the binary molten salt electrolyte are stable, and the trace impurities in the salt have no influence on the experiment. CV curve of NaCl-CaCl_2_ system with 3 wt.% TiO_2_ shows that there are four reduction peaks, a, b, c, and d, which appear in the reduction process, and one oxidation peak d’ appears in the oxidation process. The asymmetric CV curve of NaCl-CaCl_2_ system without 3 wt.% TiO_2_ and |*i_pa_*/*i_pc_*|≠1 prove that the existence of reduction was an irreversible process. According to the four reduction peaks on the CV curve, the reduction of TiO_2_ to titanium metal may be divided into four steps, which was consistent with the above thermodynamic calculation results.

**Figure 8:**
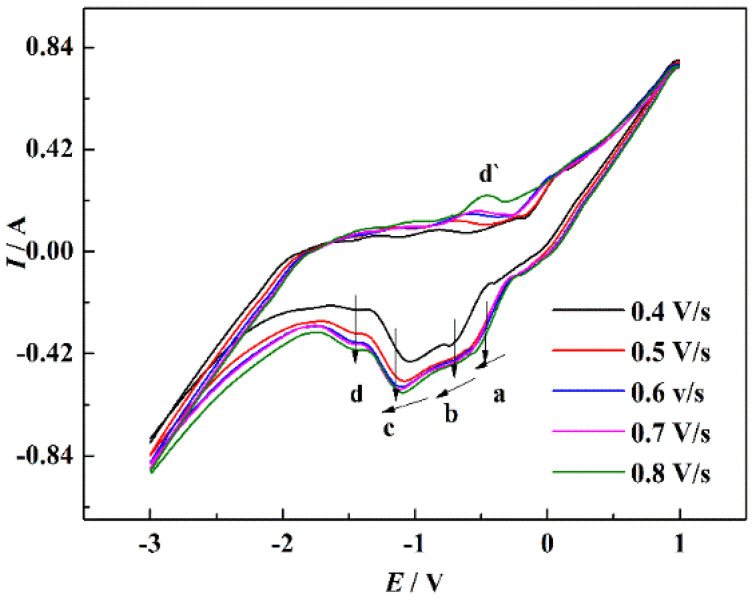
CV curves of the NaCl-CaCl_2_-TiO_2_ system with different scan rate.

**Figure 9:**
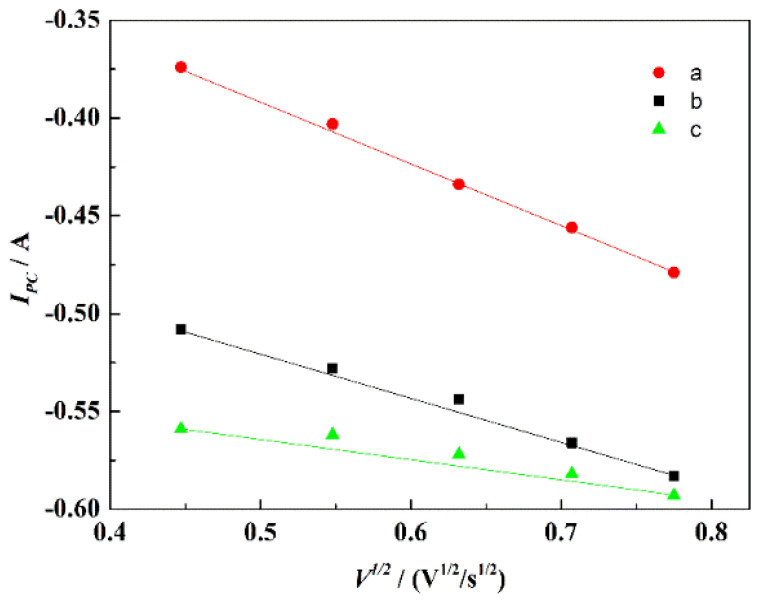
Relationship between *V^1/2^* of peaks a, b, and c and *I_pc_* in the NaCl-CaCl_2_-TiO_2_ system.

Figure 8 displays the CV curves of NaCl-CaCl_2_-TiO_2_ system with different scan rates. With the increase of the scan rate, the peak currents of the four reduction peaks gradually increased. The reduction potential corresponding to peaks a, b, and c shifted negatively with the increase of the scan rate, indicating that the reduction process was irreversible or quasi-reversible. Figure 9 displays the relationship between the scan rates of peaks a, b, and c and the peak current in NaCl-CaCl_2_-TiO_2_ system. It can be seen that the square root of the scan rate of reduction peaks a, b, and c has a linear relationship with the peak current, demonstrating that the reduction processes of a, b, and c are completely irreversible processes controlled by diffusion. The potential of peak d has no obvious deviation, so the reduction process corresponding to peak d is a reversible reaction. In consequence, both reversible and irreversible processes exist in the electrochemical reduction of TiO_2_ to titanium metal in the NaCl-CaCl_2_ binary system.

For the irreversible process of the potentiodynamic scanning, the peak potential and logarithm of scan rate has the following relation, as shown in Equation (2). When *E_pc_* and ln*v* are in a linear function, the electron transfer number (*n*) in the process can be calculated according to the slope (*k*) of the fitting curve, shown in Equation (3).

(2) $$E_{pc}=E^{\Theta }\left(\frac{RT}{\alpha nF}\right)\ln\left(\frac{RTk^{\Theta }}{\left(1-\alpha \right)nF}\right)+\left(\frac{RT}{\left(1-\alpha \right)nF}\right)\ln v$$

(3) k=RT/(1−α)nF

where *E* is the peak potential (V); R, *T*, *n*, *v*, α, and F represent the ideal gas constant (8.314 J/(mol·K)), absolute temperature (K), the electron transfer number, the scan rate (V/s), the charge transfer coefficient, and Faraday’s constant (96,485 C/mol), respectively.

**Figure 10:**
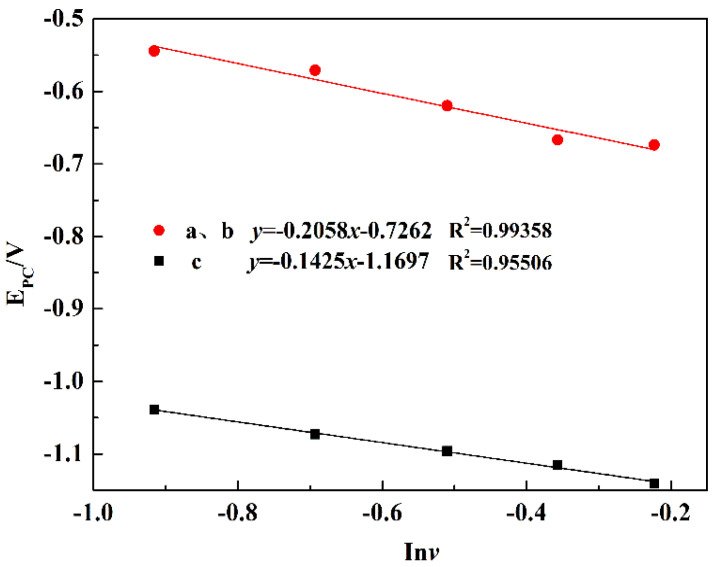
Fitting curves of the peak potential (*E_pc_*) and the logarithm scan rate (ln*v*).

According to the CV curve, the reduction potential difference of peak a and b is 0.15 V, which is consistent with the theoretical decomposition potentials difference 0.14 V of the reactions (4) and (8). Figure 10 shows the fitting curves of the peak potential (*E_pc_*) and the logarithm scan rate (ln*v*). According to the slope of the fitting line, the electron transfer number in the combined process of peaks a and b was calculated to be 1.303, approximately 1, but there were also non-stoichiometric Ti_4_O_7_ in the reduction process of TiO_2_ to Ti_2_O_3_. Due to the small theoretical decomposition potential difference, the two independent peaks a and b could be approximately regarded as one peak. Peaks a and b represent the reduction process from TiO_2_ to Ti_2_O_3_ by direct reduction or a step-by-step process with an electron transfer number of 1, and Ti_4_O_7_ reduced to Ti_2_O_3_ was also controlled by diffusion [25]. The electron transfer number of peak c was calculated to be 1.298, approximately 1. According to the electron transfer number, the diffusion coefficients of diffusion-controlled processes A, B, and C are 0.349 × 10^−5^ cm/s and 0.2352 × 10^−4^ cm/s, respectively. The formula is shown in Equation (4) [27]:

(4) $$i_{p}=0.4958nAFC_{o}{\left(\frac{\alpha nFD_{o}v}{RT}\right)}^{1/2}$$

where *i_P_* is peak current density (A/cm^2^); *C_o_*, *A*, and *D_o_* represent the concentration of the reactants (mol/cm^3^), work electrode area (1.95465 cm^2^), and diffusion coefficient (cm^2^/s), respectively.

**Figure 11:**
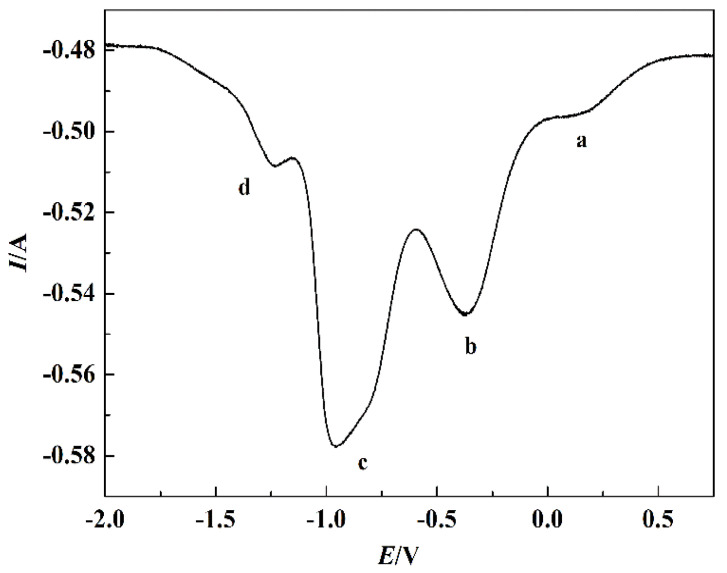
Square wave voltammetry curves of the NaCl-CaCl_2_-TiO_2_ system with 4 V/s scan rate.

Figure 11 shows the square wave voltammetry curve of the NaCl-CaCl_2_-TiO_2_ system. Three obvious reduction peaks between −1.5 V and 0 V can be seen from the curve. The first peak of process a and b is near −0.5 V, the second peak of process c is near −1.0 V, and the third peak of process d is near −1.4 V, which is roughly the same as the reduction peak potential of the CV curve. The irreversible process in the reduction process is the main reason for a little shift of the reduction peak. Process d is a reversible process, so the relationship between the half-peak width and the electron transfer number can be expressed in Equation (5) [28]. The electron transfer number in process d calculated by Equation (5) is 2.324, approximately 2, which corresponds to reaction (13). The reduction process of TiO_2_ to titanium was further confirmed as TiO_2_→Ti_4_O_7_→Ti_2_O_3_→TiO→Ti.

(5) $$E_{1/2}=3.52\left(\frac{RT}{nF}\right)$$

## 4. Conclusions

Titanium metal was prepared by the electrochemical reduction in NaCl-CaCl_2_ binary molten salt at 1073 K, and the reduction process of TiO_2_ to titanium can be summarized as TiO_2_→Ti_4_O_7_→Ti_2_O_3_→TiO→Ti. As an intermediate product in the deoxidation process of TiO_2_, CaTiO_3_ can be spontaneously generated among Ca^2+^, O^2−^, and TiO_2_ in the NaCl-CaCl_2_ system. The dissolution behavior of TiO_2_ showed that there is no chemical dissolution of TiO_2_ in the NaCl-CaCl_2_ molten salt system at 1073 K. Electro-deoxidation thermodynamics and electrochemical studies further confirmed that the reduction of TiO_2_ to titanium in four steps, and the processes were controlled by diffusion.

## Figures and Tables

**Table 2:** Δ*G*^θ^ and *E* of TiO_2_ electro-deoxidation reactions at 1073 K.

Reactions	Δ*G*^θ^_1073 K_ (kJ/mol)	*E* (V)	No.
8TiO_2_ + C = 2Ti_4_O_7_ + CO_2_ (g)	32.82	−0.34	(4)
4TiO_2_ + C = 2Ti_2_O_3_ + CO_2_ (g)	39.57	−0.41	(5)
2TiO_2_ + C = 2TiO + CO_2_ (g)	56.09	−0.58	(6)
TiO_2_ + C = Ti + CO_2_ (g)	353.86	−0.92	(7)

**Table 3:** Δ*G*^θ^ and *E* of Ti_4_O_7_, Ti_2_O_3_, and TiO electro-deoxidation reactions at 1073 K.

Reactions	Δ*G*^θ^_1073 K_ (kJ/mol)	*E* (V)	No.
2Ti_4_O_7_ + C = 4Ti_2_O_3_ + CO_2_ (g)	46.31	−0.48	(8)
Ti_4_O_7_ + 1.5C = 4TiO + 1.5CO_2_ (g)	95.76	−0.99	(9)
Ti_4_O_7_ + 3.5C = 4Ti + 3.5CO_2_ (g)	337.44	−3.50	(10)
2Ti_2_O_3_ + C = 4TiO + CO_2_ (g)	72.60	−0.75	(11)
Ti_2_O_3_ + 1.5C = 2Ti + 1.5CO_2_ (g)	314.29	−1.09	(12)
2TiO + C = 2Ti + CO_2_ (g)	241.68	−1.25	(13)

## Data Availability

Data sharing is not applicable to this article.
